# Cardiogenic Shock and Symmetrical Peripheral Gangrene in Relapsed Follicular Lymphoma: A Case Report With Bordetella bronchiseptica Co-infection

**DOI:** 10.7759/cureus.107125

**Published:** 2026-04-15

**Authors:** Keval Thakkar, Bilind Ismail, John Greene

**Affiliations:** 1 Internal Medicine, Willis Knighton Health, Shreveport, USA; 2 Internal Medicine, Moffitt Cancer Center, Tampa, USA

**Keywords:** bordetella bronchiseptica, cardiogenic shock, digital amputation, follicular lymphoma, symmetrical peripheral gangrene, vasopressor-induced ischemia, zoonotic infection

## Abstract

We present a case of a 53-year-old man with longstanding relapsed follicular lymphoma who was admitted with a febrile respiratory illness following close contact with multiple dogs and suffered a cardiac arrest on the first day of hospitalization. He was successfully resuscitated but developed an acute myocardial infarction with severely reduced ventricular function and cardiogenic shock requiring vasopressor support. The resulting peripheral hypoperfusion produced symmetrical dry gangrene of all ten digits and both feet, requiring multiple digital and bilateral foot amputations. Respiratory cultures obtained after antibiotics were held, once the patient was clinically stable, grew Bordetella bronchiseptica and Haemophilus parainfluenzae, organisms likely colonizing the respiratory tract from the time of admission. Targeted therapy with levofloxacin was completed, the patient was extubated, cardiac function recovered fully, and he was discharged with plans to resume lymphoma-directed treatment. This case highlights the vulnerability of patients with untreated relapsed hematologic malignancy to atypical zoonotic respiratory pathogens and illustrates the vasopressor-driven mechanism of symmetrical peripheral gangrene in the setting of post-arrest cardiogenic shock.

## Introduction

Bordetella bronchiseptica is a gram-negative coccobacillus best known in veterinary medicine as the principal cause of infectious tracheobronchitis, or kennel cough, in dogs. Human infection is uncommon and occurs almost exclusively in patients with significant immunosuppression, including those with hematologic malignancy, HIV infection, solid organ transplantation, or prolonged corticosteroid use [1,2]. The organism can colonize the respiratory tract of susceptible hosts following animal contact and is frequently difficult to detect on standard culture media, particularly when patients are already receiving broad-spectrum antibiotics, making the diagnosis easy to overlook and often delayed [2,3]. As of 2006, fewer than 55 cases of human Bordetella bronchiseptica infection had been reported in the literature, with the majority occurring in immunocompromised hosts, underscoring its rarity as a human pathogen [1,2].

In the immunocompromised host, respiratory infection with organisms such as Bordetella bronchiseptica can contribute to the hemodynamic deterioration that precipitates such circulatory failure.

Symmetrical peripheral gangrene is defined as ischemic necrosis of two or more distal extremities in the absence of large vessel occlusion or primary vasculitis [4]. It most commonly arises from the combination of systemic hypoperfusion, microvascular thrombosis, and vasospasm in the setting of septic or cardiogenic shock [4,5]. High-dose vasopressors used during resuscitation are a recognized independent contributor, redirecting blood flow centrally and compounding distal ischemia even as cardiac output begins to recover [5,6]. Symmetrical peripheral gangrene complicating circulatory failure is rare, with reported estimates of less than one percent of critically ill patients, but carries significant morbidity, including the risk of major limb loss [4,5].

Relapsed follicular lymphoma carries a compounded risk in this setting. Follicular lymphoma is characterized by a defective immune microenvironment that suppresses normal T-cell and natural killer cell activity, and untreated progressive disease adds systemic inflammatory burden with exhausted physiologic reserve that makes critical illness both more likely and harder to recover from [7,8]. We present a case in which these vulnerabilities converged: a patient with active, untreated lymphoma developed a febrile respiratory illness in which Bordetella bronchiseptica was later identified in respiratory cultures, suffered a cardiac arrest with myocardial injury and cardiogenic shock, and developed extensive symmetrical dry gangrene requiring amputations of multiple digits and both feet. Importantly, the peripheral gangrene in this case was a direct consequence of cardiogenic shock and vasopressor-dependent resuscitation, rather than a primary infectious complication of the respiratory illness.

## Case presentation

The patient is a 53-year-old man with a 13-year history of follicular lymphoma. At the time of his original diagnosis, the disease involved lymph nodes, spleen, and bone, and he was treated with combination chemoimmunotherapy including bendamustine, ofatumumab, and bortezomib as part of a clinical trial, achieving remission and remaining on observation for approximately a decade. He subsequently developed progressive lymphadenopathy with new cervical, inguinal, and lumbar involvement, and biopsy confirmed relapsed low-grade classical follicular lymphoma. Treatment options were discussed, and he elected observation. Over the following months, he developed worsening fatigue and drenching night sweats consistent with active B symptoms, and imaging demonstrated further disease progression, confirmed on repeat lymph node biopsy. At the time of his current presentation, he had active, untreated, progressive lymphoma with systemic symptoms and no directed therapy, rendering him functionally immunocompromised from his underlying malignancy.

He presented with a three-day history of fevers, body aches, night sweats, flank pain, sore throat, and a dry, non-productive cough, along with swollen cervical nodes, diarrhea, and tea-colored urine. He was a lifelong non-smoker. He had close contact with multiple dogs in his environment, and two young grandchildren in the household had concurrent nasal congestion and upper respiratory symptoms. On evaluation, he was febrile at 38.4 degrees Celsius with a heart rate of 110 beats per minute. White blood cell count was elevated, and procalcitonin was mildly elevated at 0.17 ng/mL, a value that may reflect early or partially treated bacterial illness, or a predominantly non-bacterial process at this early stage of presentation. Urinalysis was unremarkable. Initial chest radiograph showed no infiltrates. CT of the abdomen and pelvis demonstrated bulky lymphadenopathy consistent with his known lymphoma burden without acute abdominal findings. He was admitted for a febrile syndrome and started on ceftriaxone and azithromycin empirically. A respiratory pathogen panel was offered, but the patient declined on the basis of personal skepticism toward PCR-based testing.

**Figure 1:**
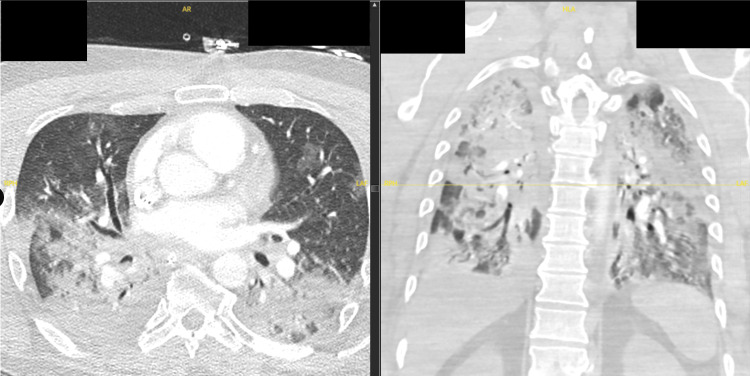
CT chest obtained after cardiac arrest and resuscitation. Axial view (left) demonstrates extensive bilateral pulmonary consolidations with a dependent and parahilar distribution, more prominent in the right lower lobe, with areas of anterior ground-glass opacity. Coronal view (right) shows the bilateral extent of the consolidative and ground-glass changes. Findings were consistent with pulmonary edema in the setting of acute cardiac dysfunction with superimposed consolidation treated clinically as pneumonia of unknown etiology.

On the first day of hospitalization, he suffered a pulseless electrical activity cardiac arrest. Cardiopulmonary resuscitation was performed, and return of spontaneous circulation was achieved. He was emergently intubated for acute respiratory failure. Troponin peaked at approximately 1,500 ng/L, consistent with an acute myocardial infarction, and echocardiography demonstrated severely reduced left ventricular function with an ejection fraction of 25 to 30 percent. The clinical picture was consistent with cardiogenic shock from post-arrest myocardial stunning compounded by ischemic injury. The precise trigger of the arrest was not definitively established, though potential contributors included hypoxemia from the underlying respiratory illness, demand ischemia in the setting of systemic inflammation and tachycardia, sepsis-related myocardial dysfunction, and the small pulmonary embolism identified on subsequent imaging. Vasopressors were initiated to maintain hemodynamic stability. Post-arrest CT of the chest showed extensive bilateral pulmonary consolidations in the dependent and parahilar regions with areas of anterior ground-glass opacity and new small bilateral pleural effusions, along with a small non-occlusive subsegmental pulmonary embolism (Figure 1). The imaging findings were consistent with pulmonary edema in the setting of acute cardiac dysfunction, and the clinical team also treated the consolidation as pneumonia of unknown etiology, given the overall presentation. New anterior rib fractures were attributed to resuscitation. Antibiotics were escalated to vancomycin and cefepime and subsequently to meropenem, given the severity of illness and absence of a microbiologic diagnosis.

Bronchoscopy with bronchoalveolar lavage performed shortly after intubation returned culture negative for significant bacterial pathogens, with only environmental fungal colonization noted. A subsequent sputum culture while the patient remained on meropenem showed four-plus usual respiratory flora and was non-diagnostic. Meropenem was continued for seven days and then held once the patient was clinically stable. A repeat sputum culture obtained after antibiotics were held grew Bordetella bronchiseptica at one-plus on semi-quantitative culture, along with three-plus Haemophilus parainfluenzae (beta-lactamase negative) and four-plus usual respiratory flora. Susceptibility testing of the Bordetella bronchiseptica isolate demonstrated sensitivity to doxycycline, intermediate susceptibility to piperacillin-tazobactam, and resistance to trimethoprim-sulfamethoxazole. Levofloxacin was initiated for a seven-day course, given its activity against both isolated organisms. The patient was successfully extubated during this period, and his respiratory status improved steadily.

**Figure 2:**
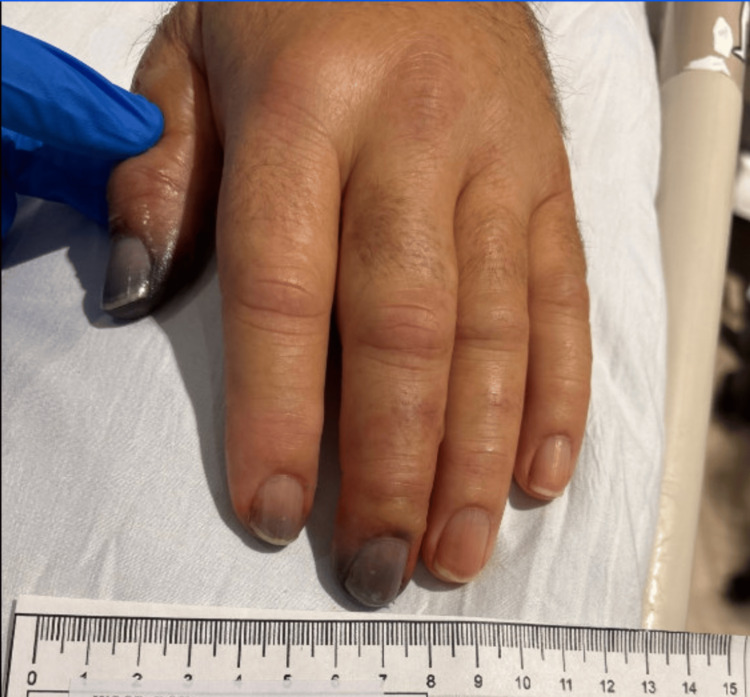
Left hand demonstrating early ischemic changes following cardiogenic shock and vasopressor-dependent resuscitation. The thumb shows the most advanced discoloration with dark mummification of the distal tip. The Index and middle fingers demonstrate early darkening at the nail beds with beginning demarcation between ischemic and viable tissue.

**Figure 3:**
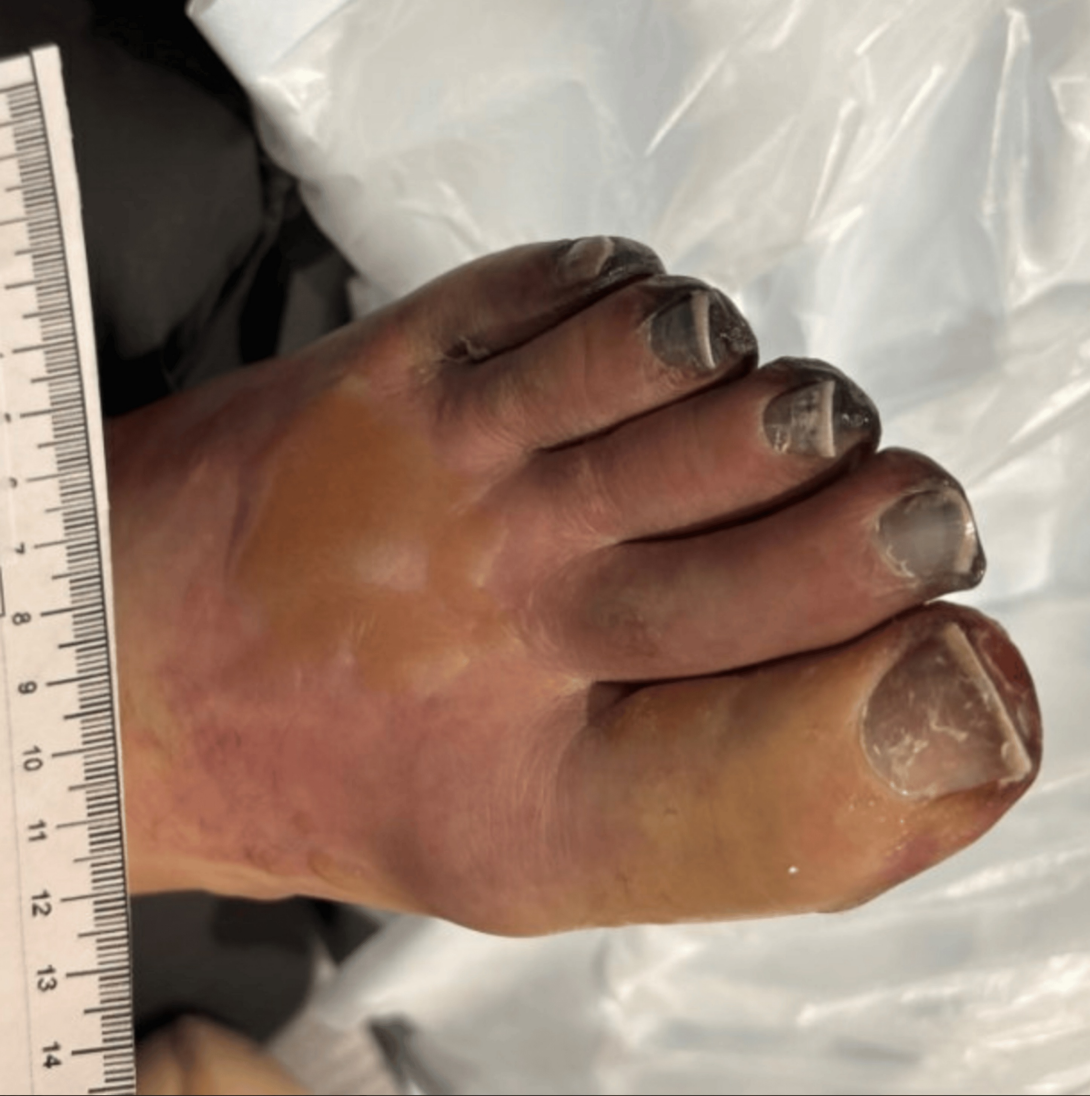
Left foot demonstrating advanced symmetrical dry gangrene of all five toes following cardiogenic shock. Well-demarcated black mummification is present distally across all digits with sharp demarcation from viable tissue proximally.

Throughout the period of cardiogenic shock and vasopressor dependence, progressive darkening and demarcation developed across all ten digits bilaterally and both feet. The thumb and distal fingers of the left hand showed early dark discoloration with visible demarcation between ischemic and viable tissue (Figure 2). All toes of the left foot demonstrated well-advanced dry black necrosis with complete distal mummification and sharp demarcation, without surrounding erythema, warmth, or purulent discharge (Figure 3). The right hand and right foot showed comparable changes. Vascular surgery was consulted and managed the patient expectantly, allowing full tissue demarcation before any surgical decision was made. The patient ultimately underwent multiple digital amputations and bilateral tarsometatarsal amputations. Pathologic examination of the amputated digit specimens demonstrated skin and soft tissue necrosis with acute and chronic inflammation, focal erosion into adjacent bone, and hypocellular bone marrow without significant inflammatory infiltrate, consistent with ischemic necrosis from peripheral hypoperfusion rather than primary infectious invasion of the tissue. Renal function, which had deteriorated during the shock state, recovered over the course of the hospitalization. Ejection fraction normalized to 60 to 65 percent on follow-up imaging. He was discharged and, at subsequent follow-up, had healing amputation sites, no active infection, and was being evaluated for resumption of lymphoma-directed therapy.

## Discussion

The primary illness in this case was non-Hodgkin lymphoma complicated by acute myocardial injury, heart failure, and cardiogenic shock. The dry gangrene was a direct consequence of that shock state and the vasopressor dependence it required. Bordetella bronchiseptica was identified later in the course as a contributor to the respiratory illness, after antibiotics were held once the patient was clinically stable and cultures could become meaningful. These three threads, the underlying lymphoma, the cardiac event, and the respiratory infection, each played a role in this patient’s illness, but they did not carry equal weight, and the manuscript should not imply otherwise.

The patient’s lymphoma history is essential context. This was not incidentally discovered indolent disease. He had a 13-year course with documented multi-site involvement at diagnosis, prior chemoimmunotherapy, a period of remission, and then progressive relapse that had gone untreated for over a year before this presentation. He had active B symptoms and worsening systemic disease burden. Follicular lymphoma is characterized by a defective immune microenvironment that impairs both T-cell and humoral immune function, and this immunologic vulnerability is present even without active cytotoxic therapy [7,8]. Untreated relapse compounds this deficit further. It was this accumulated immune impairment that created the conditions for Bordetella bronchiseptica to colonize and subsequently infect the respiratory tract, an event that rarely occurs in immunocompetent individuals [1,2].

The epidemiologic source in this case is clinically important. The patient had close contact with multiple dogs, the primary animal reservoir of Bordetella bronchiseptica, which can shed the organism asymptomatically without any visible signs of kennel cough [1,2]. Young grandchildren in the household with concurrent upper respiratory symptoms may have shared the same animal exposure or served as a secondary link in the household transmission chain, though neither route could be confirmed without contact testing [3]. What the exposure history establishes is a biologically plausible and well-recognized route of acquisition. The organism most likely colonized the patient’s respiratory tract at or near the time of his presentation, consistent with the culture data showing low-burden growth that only appeared after antibiotics were held.

The microbiologic workup in this case illustrates a pattern that clinicians should recognize. Bordetella bronchiseptica grows slowly, requires selective or enriched media for optimal recovery, and is reliably suppressed by concurrent broad-spectrum antibiotic therapy [2,3]. Three sequential cultures were needed before the organism declared itself: the initial bronchoalveolar lavage was negative, the early sputum culture showed only usual respiratory flora while the patient was on meropenem, and only the culture obtained after antibiotics were held yielded the organism. Holding antibiotics once a patient is clinically stable, when standard empiric regimens have not produced a microbiologic answer, is a legitimate and sometimes necessary diagnostic step in the immunocompromised host [2]. The co-isolation of Haemophilus parainfluenzae at three-plus burden should not be dismissed as simple colonization. At that level of growth, in a significantly immunocompromised patient with active lower respiratory tract illness, it likely represented a contributing pathogen, and both organisms were covered effectively by levofloxacin [9]. The role of the initial respiratory illness in the chain of events leading to cardiac arrest is uncertain and cannot be determined from the available data. What can be said is that a febrile illness in a host with exhausted physiologic reserve and active lymphoma contributed to the overall clinical deterioration. While hypoxemia, demand ischemia, sepsis-related myocardial dysfunction, and the small pulmonary embolism identified on imaging were each considered as potential contributors to the arrest, definitive attribution to any single etiology was not possible, given the clinical complexity.

Symmetrical peripheral gangrene arising from shock has been associated with a range of bacterial pathogens. Among gram-negative organisms, Neisseria meningitidis is the classic cause of purpura fulminans and disseminated intravascular coagulation [4,5]. Escherichia coli in urosepsis, Pseudomonas aeruginosa, and Klebsiella pneumoniae in gram-negative bacteremia, and among gram-positive pathogens, Group A Streptococcus, Streptococcus pneumoniae, and Staphylococcus aureus have each been documented as causes of shock-driven acral ischemia [4,5]. Regardless of the trigger, the final pathway is microvascular thrombosis and critically reduced distal perfusion. In this case, the mechanism was primarily cardiogenic: an ejection fraction of 25 to 30 percent with vasopressor-dependent resuscitation drove the peripheral hypoperfusion, and multiple clinical services attributed the digital ischemia explicitly to vasopressor use in the context of cardiogenic shock [5,6]. The pathologic findings of ischemic necrosis with hypocellular, non-inflamed bone marrow in the amputated specimens confirm this mechanism and establish the absence of primary infectious invasion of the digit tissue. The etiology of the cardiac arrest itself, while not definitively established, likely reflected a combination of factors including hypoxemia from the respiratory illness, demand ischemia in the setting of tachycardia and systemic inflammation, and possible contribution from the small pulmonary embolism identified on imaging.

The management of dry gangrene following cardiogenic shock follows a staged approach with restoring systemic perfusion first, then allowing the ischemic tissue to fully demarcate before any surgical intervention [10]. Dry and well-demarcated necrosis without surrounding infection is managed expectantly, as premature amputation risks entering viable tissue with poor wound healing consequences. Vascular surgery consultation guides timing, amputation level, and interim wound care [10]. Anticoagulation may be considered when ongoing microvascular thrombosis appears to be propagating ischemia, though supporting evidence is limited [6]. In this patient, full demarcation was awaited before surgical planning, and the pathologic confirmation of ischemic rather than infectious tissue loss guided the decision to limit antibiotic therapy to the respiratory indication alone. This case also raises important questions for future research regarding optimal vasopressor thresholds in immunocompromised patients with limited physiologic reserve, and the role of prophylactic counseling regarding zoonotic infection risk in patients with hematologic malignancy who have regular animal contact.

## Conclusions

This case documents an uncommon combination of events in a patient whose longstanding, untreated follicular lymphoma created the immunologic conditions for everything that followed. Bordetella bronchiseptica, most likely acquired through close contact with multiple dogs, was a contributing respiratory pathogen identified only after antibiotics were held once the patient was clinically stable. The cardiac arrest, acute myocardial injury, and cardiogenic shock were the primary drivers of the symmetrical dry gangrene, with high-dose vasopressor use compounding peripheral ischemia during resuscitation. Serial respiratory cultures and a period of antibiotic de-escalation were essential to the microbiologic diagnosis, and levofloxacin provided effective targeted coverage. Despite the severity of his illness and the extent of his amputations, the patient survived with fully recovered cardiac function and was positioned to resume lymphoma-directed therapy. Clinicians managing immunocompromised patients with active hematologic malignancy should consider Bordetella bronchiseptica in the respiratory differential following animal contact, pursue serial microbiologic sampling when empiric regimens are failing, and approach vasopressor-associated peripheral gangrene with an expectant surgical strategy guided by full tissue demarcation. More broadly, this case supports the need for heightened clinical awareness of zoonotic respiratory pathogens in immunocompromised patients with animal contact, and suggests that institutional guidelines for this population should include structured inquiry into animal exposure as part of the infectious disease workup.
